# Supplementary material for: The Mechanism of Action of L-Tyrosine Derivatives against Chikungunya Virus Infection In Vitro Depends on Structural Changes
Source: Int J Mol Sci. 2024 Jul 21;25(14):7972. doi: 10.3390/ijms25147972 (PMC11277544; doi:10.3390/ijms25147972)
Supplement: Supplementary file 1 [file ijms-25-07972-s001.zip › Supplementary Figure S3 (20-07-2024).pdf]

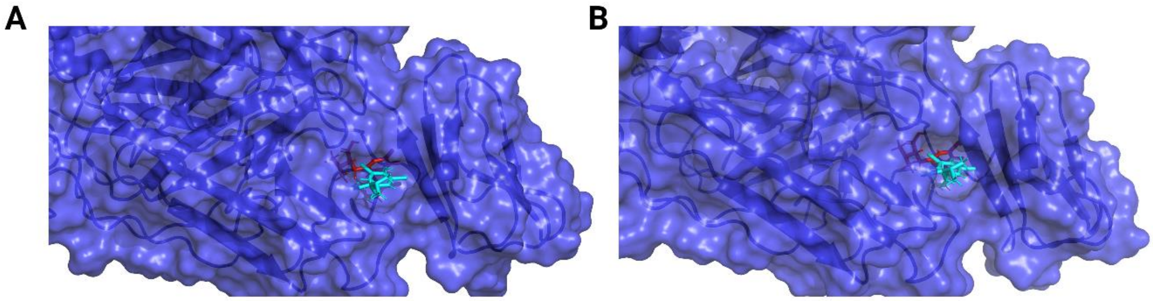

Supplementary Figure S3. Topologies of the 3N42-L-tyrosine derivatives complexes. A) Complex 3N42 with compound 2. B) Complex 3N42 with compound 3. The cyan structure represents the topology at 0 ns, while the red structure represents the topology at 100 ns.
