# Supplementary figures and images for: The Mechanism of Action of L-Tyrosine Derivatives against Chikungunya Virus Infection In Vitro Depends on Structural Changes
Source: Int J Mol Sci. 2024 Jul 21;25(14):7972. doi: 10.3390/ijms25147972 (PMC11277544; doi:10.3390/ijms25147972)

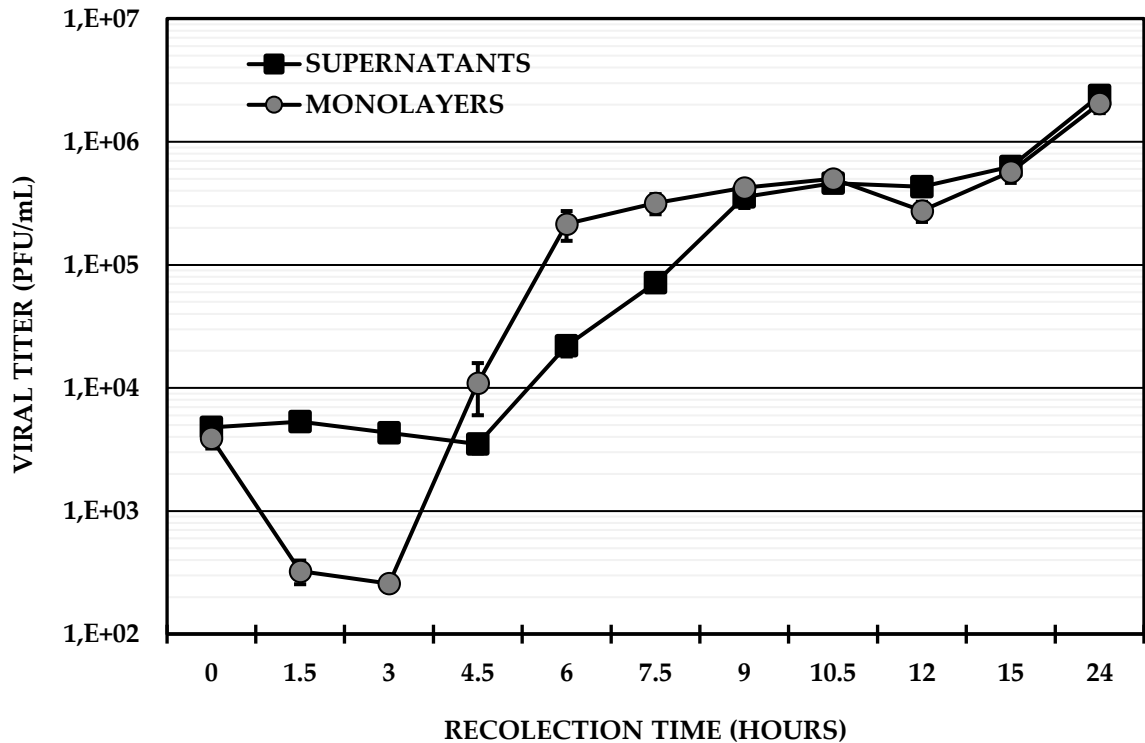

**Supplementary Figure S1. CHIKV/Col 24 h replication curve in Vero cells.**

Supplement: Supplementary file 1 [file ijms-25-07972-s001.zip › Supplementary Figure S1 (20-07-2024).pdf]

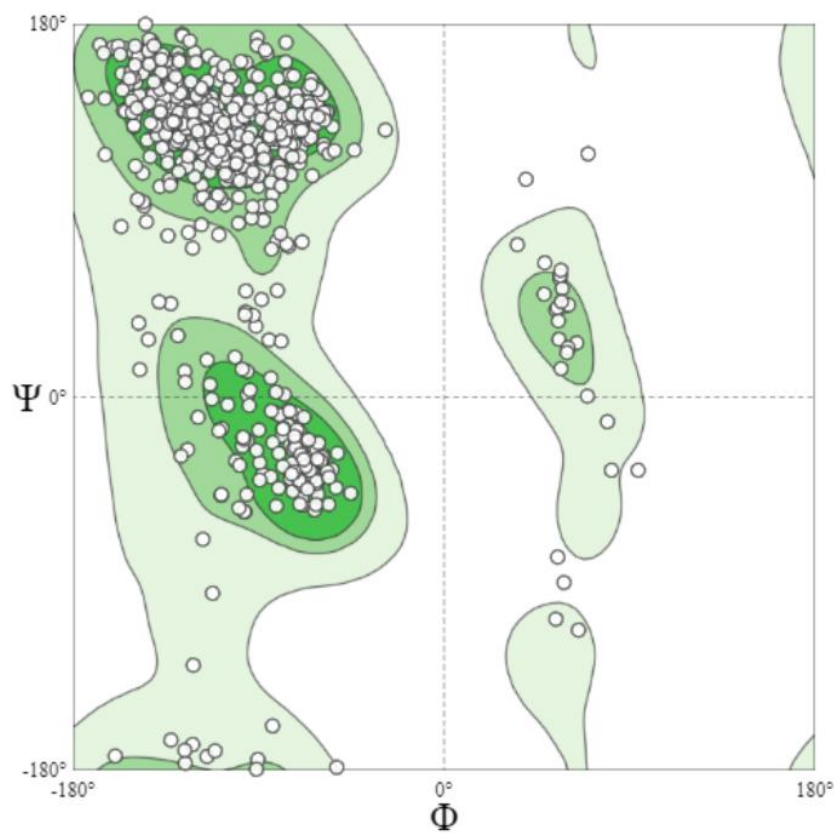

Supplementary Figure S2. Ramachandran plot for assessing the quality of the protein 3N42 .

Supplement: Supplementary file 1 [file ijms-25-07972-s001.zip › Supplementary Figure S2 (20-07-2024).pdf]
